# Mutual interactions of the apicomplexan parasites *Toxoplasma gondii* and *Eimeria tenella* with cultured poultry macrophages

**DOI:** 10.1186/s13071-018-3040-0

**Published:** 2018-08-06

**Authors:** Runhui Zhang, Ahmed Thabet, Lysanne Hiob, Wanpeng Zheng, Arwid Daugschies, Berit Bangoura

**Affiliations:** 1 Institute of Parasitology, Centre for Infectious Diseases, Leipzig University, Leipzig, Germany; 2 TCVS diagnostic laboratory-Gaza, Gaza strip, Palestine; 3 Department of Veterinary Sciences, University of Wyoming, Laramie, WY USA; 4 Albrecht-Daniel-Thaer-Institute, Leipzig, Germany

**Keywords:** *Toxoplasma gondii*, *Eimeria tenella*, Co-infection, *in vitro*, Chicken macrophage

## Abstract

**Background:**

*Toxoplasma gondii* and *Eimeria tenella* are two common parasites in poultry. Mixed infections are likely to occur frequently in chickens due to the high prevalence of both pathogens. In this study, we investigate the co-occurrence of the two pathogens in the same immunocompetent host cell population towards potential parasite-parasite as well as altered patterns of parasite-host interactions.

**Methods:**

Primary macrophages from chicken blood were co-infected *in vitro* with *T. gondii* tachyzoites (RH strain) and *E. tenella* sporozoites (Houghton strain) for 72 h. Morphological observations by light microscopy and assessments of parasite replication by quantitative real-time PCR (qPCR) were performed at 24, 48 and 72 h post-infection (hpi). Six host cell immune factors previously linked to *T. gondii* or *E. tenella* infection were selected for gene expression analysis in this study.

**Results:**

Distinct morphological changes of macrophages were observed during mixed infection at 24 hpi and immunological activation of host cells was obvious. Macrophage mRNA expression for iNOS at 48 hpi and for TNF-α at 72 hpi were significantly elevated after mixed infection. Distinct upregulation of IL-10 was also present during co-infection compared to *Eimeria* mono-infection at 48 and 72 hpi. At 72 hpi, the total number of macrophages as well as the number of both parasites decreased markedly. As measured by qPCR, *E. tenella* population tended to increase during *T. gondii* co-infection, while *T. gondii* replication was not distinctly altered.

**Conclusions:**

Mutual interactions of *T. gondii* and *E. tenella* were observed in the selected co-infection model. The interactions are supposed to be indirect considering the observed changes in host cell metabolism. This study would thus help understanding the course of co-infection in chickens that may be relevant in terms of veterinary and zoonotic considerations.

## Background

*Toxoplasma gondii* and *Eimeria tenella* are two coccidian parasites in poultry. *Toxoplasma gondii* is a heteroxenous parasite and may inhabit a wide range of vertebrate species as intermediate host, which may harbor the cyst stages in various tissues [1] following tachyzoite replication*.* Several reports showed high seroprevalence of *T. gondii* in chickens worldwide [2–4]. In contrast, *E. tenella* is very host-specific and monoxenous, infecting particularly the caecal mucosa of chicken [5, 6].

Mixed infections are likely to occur in chickens, particularly under free-ranging conditions. Many studies investigate host-pathogen interactions of single *T. gondii* or *Eimeria* spp. infections [7–9], but little is known to date about the pathogen-pathogen-host cell interactions for simultaneous co-infections with species of these two parasite genera. Tachyzoites of *T. gondii* and merozoites of *Eimeria* differ in many features; however, asexual stages of these parasites share ultrastructural similarities [10, 11]. It was shown that transgenic *E. tenella* (Et-TgSAG1) may induce certain immunoprotection against *T. gondii* [12]. On the other hand, *T. gondii* as a vaccine vector has a partially protective effect against coccidiosis [13]. Mixed infections with *T. gondii* and *Eimeria* spp. were found in wild rabbits in a recent case report [14]. The ability of *T. gondii* to suppress the macrophage-associated defense to *Mycobacterium avium* has been shown *in vitro* [15].

Chicken macrophages are crucially involved in the host immune response to both *T. gondii* and *E. tenella* [2, 16, 17]. In coccidiosis, chicken macrophages are also involved in sporozoite transport during the endogenous phase of *Eimeria* development [18]. Thus, it appears likely that simultaneous appearance of these two apicomplexan parasites may affect the reaction of host macrophages to parasite infection. Experimental *in vivo* studies revealed a partial mutual interaction between *T. gondii* and *E. tenella* [19] in chickens.

Immune response of primary monocyte-derived macrophages is stimulated *in vitro* by *T. gondii* tachyzoites [20] and *E. tenella* sporozoites [21]. High expression of pro-inflammatory Th1 cytokines is typically related to macrophage function in *T. gondii* infection [22]. Previous investigations also showed that Th1/Th2 pro-inflammatory cytokines related to macrophages are involved in the host response to *Eimeria* infection [23–25]. High production of Th2 inflammatory mediators such as interleukin 6 (IL-6) was reported in *T. gondii* and *E. tenella* mono-infections [26, 27]. *In vitro* replication of *T. gondii* is enhanced significantly when murine macrophages are pre-treated with IL-6 prior to infection [28]. *In vitro* infection of avian macrophages by *E. tenella* sporozoites upregulates nitric oxide (NO) production and inducible nitric oxide synthase (iNOS) transcription [29, 30]. Induced NO production by macrophages is generally related to cytokines such as IFN-γ and tumor necrosis factor α (TNF-α) which play a vital role in immunity of chicken against coccidiosis and toxoplasmosis [31, 32]. Various effects of *in vivo* co-infection of *T. gondii* and *E. tenella* in chickens [19] were observed on IFN-γ, TNF-α, IL-10 and IL-12.

In this study, we aimed to understand the simultaneous co-occurrence of the two pathogens in the same avian immune-competent host cell population and the interaction with the innate immunity against each single pathogen. Additionally, pathogen-pathogen interactions in terms of invasion and replication potential were investigated. The ability of macrophages to host both parasites simultaneously is also demonstrated.

## Methods

### Macrophages and parasites

Macrophages were separated and collected from chicken peripheral blood mononuclear cells (PBMC) according to established protocols [33]. The isolated PBMC (5 × 10^6^ cells per well) were suspended in RPMI-1640 medium (Sigma, Taufkirchen, Germany) supplemented with 5% chicken serum and 5% fetal bovine serum, penicillin (100 U/ml, PAA), streptomycin (0.1 mg/ml, PAA), and amphotericin B (0.0025 mg/ml, PAA), seeded into 24-well plates and incubated at 41 °C with 5 % CO_2_. They were grown to about 90% confluence within 96 h cultivation time. Free transgenic *T. gondii* RH-GFP tachyzoites (type I strain, kindly provided by Professor Dominique Soldati-Favre, University of Geneva Medical School, Switzerland) were harvested from infected human foreskin fibroblast (HFF) cultures by mechanical destruction. *Eimeria. tenella* Houghton strain (kindly provided by Professor Damer Blake, Royal Veterinary College, UK) and transgenic Houghton-YFP strain (kindly provided by Professor Xun Suo, China Agricultural University, China) sporozoites were gained by oocyst excystation following an established protocol [34]. Briefly, the oocyst wall of *E. tenella* was destroyed mechanically with 0.5 mm glass beads (BioSpec Products, Bartlesville, OK, USA). Excystation of sporozoites was performed by incubation with 0.25% trypsin (w/v) (Carl Roth, Karlsruhe, Germany) and 4% sodium taurocholic acid (w/v) (Sigma-Aldrich, Taufkirchen, Germany) at 41 °C for 90 min. Purified sporozoites were collected by passage through columns of nylon wool and DE-52 resin (Whatman, GE Healthcare, USA) with 1% glucose phosphate-buffered saline (PBS) at pH 7.6 (follow buffer). *E. tenella* Houghton strain sporozoites were used for all experiments except for laser scanning analyses where *E. tenella* Houghton-YFP strain was utilized.

### Mono- and co-infections

Infection doses for both parasites were optimized prior to the co-infection trial (data not shown). Six groups were set-up for *in vitro* infection studies in primary macrophages: TH, *T. gondii* infection with 5 × 10^5^ tachyzoites (high-dose); TL, *T. gondii* infection with 2.5 × 10^5^ tachyzoites (low-dose); EH, *E. tenella* infection with 5 × 10^5^ sporozoites (high-dose); EL, *E. tenella* infection with 2.5 × 10^5^ sporozoites (low-dose); CI (co-infection), mixed infection with *T. gondii* 2.5 × 10^5^ tachyzoites and *E. tenella* 2.5 × 10^5^ sporozoites; NC, uninfected negative control cell cultures. At 96 h after isolation of PBMCs, purified primary macrophages were infected with parasites according to their group and incubated at 41 °C. At 12 hours post-infection (hpi), the wells were rinsed once with PBS to remove extracellular sporozoites. The medium was changed and cell cultures were further incubated at 41 °C. The course of infection was monitored until 72 hpi.

### Positive control MDBK culture infection

In addition to the primary macrophages, Madin-Darby Bovine Kidney (MDBK) cell line cultures were used. In those cell cultures, six infection groups were formed (TH, TL, EH, EL, CI and NC) as described before. They were carried along in parallel to macrophage trials as controls for parasite replication analysis without macrophage-specific influence on parasite-parasite interaction. MDBK cultures were maintained and infected at 41 °C to enable comparison to primary chicken macrophage cultures under comparable incubation conditions. MDBK host cell cultures were tested before to be easily maintained and multiplied at this temperature (data not shown).

### Assessments of parasite replication

Parasite replication was assessed by two parameters: microscopic examination over time and parasite-specific real-time quantitative PCR (qPCR). Morphological differences and parasite population densities were visualized in all groups at 2, 24, 48 and 72 hpi by light microscopy. For qPCR, samples from each group were collected at 24, 48 and 72 hpi. DNA extraction was carried out using the QIAamp DNA Mini Kit® (Qiagen, Hilden, Germany) according to the manufacturer’s protocol for cell cultures. The *T. gondii*-specific 529-bp repeat element was used to detect replication in a probe-based qPCR [35]. Standard curve samples were generated by gradient 10-fold dilutions of 10^7^ tachyzoites to obtain absolute DNA copy numbers for *T. gondii* amplification. Replication of *E. tenella* was measured by ITS1 fragment quantification in a SYBR Green-based PCR as described before [36]. pSCA-17 plasmid standard dilutions were prepared as measure for the relative copy number of *E. tenella* DNA as described by Thabet et. al. [37]. The qPCRs were carried out in a Stratagene MX3000P cycler (Stratagene, La Jolla, USA). The cycling program for *T. gondii* detection included 95 °C for 15 min (initial denaturation), followed by 40 cycles of 95 °C for 15 s (denaturation), 60 °C for 1 min (annealing), and 72 °C for 15 s (extension). The cycling program for *E. tenella* was performed as follows: 95 °C for 5 min (initial denaturation), followed by 40 cycles of 95 °C for 30 s (denaturation), 62 °C for 20 s (annealing), and 72 °C for 20 s (extension). For *E. tenella*, a subsequent melting curve analysis (95 °C for 1 min, 62 °C for 30 s and 95 °C for 30 s) was applied to create the dissociation curve and ensure amplicon consistency. Data represent the mean of three replicates with an acceptable standard deviation for Ct values of less than 0.5.

### Immune fluorescence assay (IFA) and confocal laser scanning microscopy (CLSM)

In addition to light microscopy, CLSM (TCS-SP8, Leica, Bensheim, Germany) was applied to observe the morphology of the infected cell cultures, for localization of parasites within the host cells, and to estimate the extent of intracellular replication. Therefore, 2 × 10^6^ PBMC per well were cultivated in 8-well chamber slides (Ibidi, Martinsried, Germany) for 4 days to grow pure cultures of primary macrophages. Infection conditions were the same as mentioned above for all infection groups (TH, TL, EH, EL, CI and NC). For CLSM, cultures were fixed with methanol for 10 min before further processing. 4', 6-diamidino-2-phenylindole (DAPI, Sigma-Aldrich, USA) was used to stain cell nuclei. Cell imaging was carried out by Leica Application Suite X (LAS X, Leica Microsystems, Wetzlar, Germany).

### Cytokine analysis

Samples were collected at 24, 48 and 72 hpi, and stored at -80 °C. Six cell culture replicates per infection group were analyzed for each time point. RNA was extracted using the RNeasy® Mini Kit (Qiagen, Hilden, Germany) following the manufacturer’s instructions. Total RNA was measured using a NanoPhotometer NP80 (Implen, Munich, Germany). The complementary DNA (cDNA) was synthesized using the Revert-Aid® first strand cDNA synthesis kit (Thermo Fisher Scientific, Darmstadt, Germany), according to the manufacturer’s instructions. Briefly, 10 ng/μl total RNA were combined with 4 μl 5× reaction buffer, 1 μl RiboLock RNase Inhibitor (20U/μl), 2 μl 10mM dNTP Mix, 1 μl RevertAid M-MuLV RT (200U/μl), 1 μl of Oligo dT Primer (100 μM) and RNase-free water to a total volume of 20 μl. The mixture was incubated at 42 °C for 60 min and the reaction was terminated by heating at 70 °C for 5 min.

The mRNA expression levels of chicken host cell cytokines were measured by reverse transcription quantitative PCR (RT-qPCR). Specific sequences of the primers for TNF-α, IL-6, IL-10, IL-12, IFN-γ and iNOS cDNA amplification were selected (Table 1). Data normalization was performed based on the two chicken cell housekeeping genes glyceraldehyde 3-phosphate dehydrogenase (GAPDH) and glucose-6-phosphate dehydrogenase (G6PDH) as described before [38]. For qPCR assay, 10 μl SYBR Green master mix (Thermo Fisher Scientific, Germany), 6.6 μl water, 0.4 μl ROX solution, 2 μl cDNA template, 0.5 μl forward primer and 0.5 μl reverse primer were used per reaction. The cycling program included 95 °C for 10 min (initial denaturation), followed by 40 cycles of 95 °C for 30 s (denaturation), 60 °C for 30 s (annealing), and 72 °C for 1 min (extension). A subsequent melting curve program (95 °C for 1 min, 55 °C for 30 s and 95 °C for 30 s) was applied to create the dissociation curve for each PCR run.

**Table 1:** Sequence of the primers used for chicken cytokine analysis by RT-qPCR

RNA target	Accession No.	Primer sequences (5'-3')		Reference
		Forward	Reverse	
GAPDH	K01458	GGTGGTGCTAAGCGTGTTAT	ACCTCTGTCATCTCTCTCCACA	[38]
G6PDH	AI981686	CGGGAACCAAATGCACTTCGT	CGCTGCCGTAGAGGTATGGGA	[64]
IFN-γ	Y07922	AGCTGACGGTGGACCTATTATT	GGCTTTGCGCTGGATTC	[38]
IL-6	AJ309540	CAAGGTGACGGAGGAGGAC	TGGCGAGGAGGGATTTCT	[38]
IL-10	AJ621614	CGGGAGCTGAGGGTGAA	GTGAAGAAGCGGTGACAGC	[38]
IL-12	NM_213571	AGACTCCAATGGGCAAATGA	CTCTTCGGCAAATGGACAGT	[38]
TNF-ɑ	AY765397.1	CTTCTGAGGCATTTGGAAGC	ACTGGGCGGTCATAGAACAG	[65]
iNOS	U46504	TGGGTGGAAGCCGAAATA	GTACCAGCCGTTGAAAGGAC	[38]

### Statistics

Relative parasite DNA copy numbers as derived from qPCR results were calculated and presented as mean value (*n* = 3) for each time point after infection. Cytokines were quantified from RT-qPCR results using qBase Plus 2.3 (Biogazelle NV, Belgium). Reference target stability and cytokine X-fold change differences between the infection groups were analyzed. X-fold change of cytokine expression was also calculated in comparison to the NC group data for each respective time point. Non-parametric Kolmogorov-Smirnov test was performed to test for normal distribution of data. Thereafter, the cytokine data were analysed statistically by ANOVA followed by Student’s t-test (SPSS version 20 ®, IBM, New York, USA). Data are presented as mean ± standard error of the mean (SEM). A *P-*value < 0.05 is considered as statistically significant.

## Results

### Visual observation by light microscopy and CLSM

**Fig. 1:**
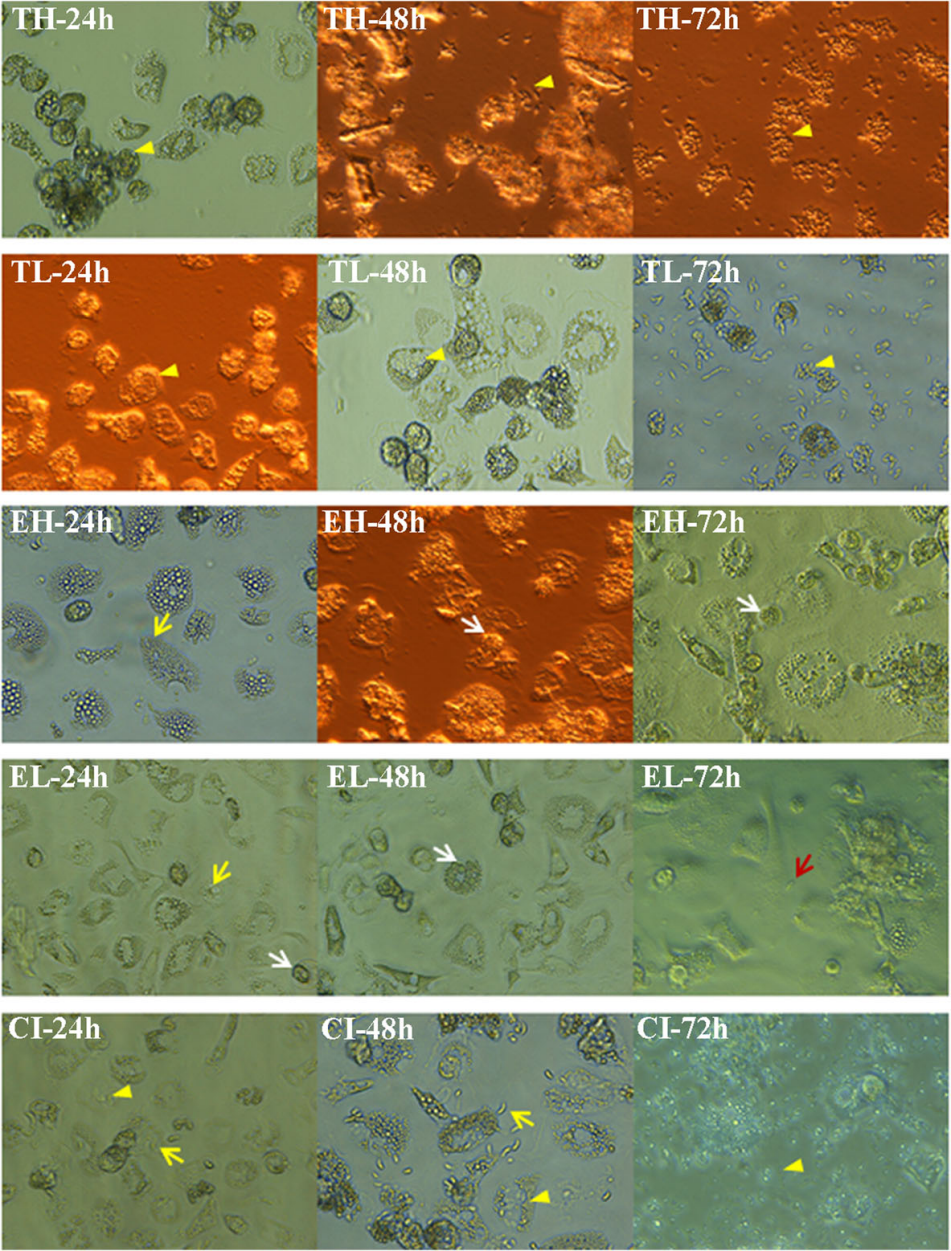
Morphological visualization of primary macrophage cell cultures at 24, 48, 72 hpi by light microscopy. Yellow arrowhead: *T. gondii* tachyzoites; yellow arrow, *E. tenella* sporozoites; white arrow, *E. tenella* meronts; red arrow: *E. tenella* merozoites. *Abbreviations*: TH, high-dose infection with *T. gondii*; TL, low-dose infection with *T. gondii*; EH, high-dose infection with *E. tenella*; EL, low-dose infection with *E. tenella*; CI, co-infected group. Negative control group (NC) not shown

All infection groups were examined by light microscopy at four time points after infection (Fig. 1 and Table 2). At 2 hpi, invasion and adherence of *E. tenella* sporozoites were more obvious than for *T. gondii* tachyzoites in mono- and co-infected cell cultures (data not shown). Macrophage vacuolization occurred 24 hpi in the CI and *Toxoplasma* groups (TL and TH), but to a lesser extent in the *Eimeria* groups (EL and EH). Meanwhile, most of the *E. tenella* sporozoites remained in the intracellular sporozoite stage with low numbers of meronts appearing in both *Eimeria*-infected groups (EL and EH) and the CI group. At 48 hpi, many macrophages were detached in the infected cultures of TH group and CI group. There was a large number of free tachyzoites in the TH group with microscopic findings similar to 72 hpi in TL group and CI group. However, less free tachyzoites were visualized in TL and CI groups at 48 hpi. After 72 h, low numbers of *E. tenella* second generation of merozoites were found in the mono-infection groups EL and EH occasionally. By light microscopy, lowest numbers of macrophages were counted at 48 hpi. Almost no intact-appearing macrophage could be observed in *T. gondii* infection groups and co-infected group CI at 72 hpi.

**Table 2:** Light microscopy findings by infection group and time point compared to uninfected NC group

	2 hpi			24 hpi			48 hpi			72 hpi		
	**EH**	**EL**	**CI**	**EH**	**EL**	**CI**	**EH**	**EL**	**CI**	**EH**	**EL**	**CI**
Attached macrophages	+++	+++	+++	+++	+++	++	+++	+++	++	++	++	+
Macrophage vacuolization	-	-	+	++	+	++	+	+	+++	++	++	+++
Free Et^a^ sporozoites	+	+	+	-	-	-	-	-	-	-	-	-
Intracellular Et ^a^ sporozoites	+	+	+	+	+	+	-	-	-	-	-	-
Intracellular meronts	-	-	-	+	+	++	++	++	++	++	++	++
Et ^a^ merozoites	-	-	-	-	-	-	-	-	-	+	+	-^c^
	**TH**	**TL**	**CI**	**TH**	**TL**	**CI**	**TH**	**TL**	**CI**	**TH**	**TL**	**CI**
Attached macrophages	+++	+++	+++	++	+++	++	+	++	++	-	+	+
Macrophage vacuolization	++	+	+	++	+	++	+++	++	+++	-	+++	+++
Free Tg^b^ tachyzoites	+++	++	++	+	-	-	+++	+	+	+	+++	+++
Intracellular Tg^b^ tachyzoites	+	+	+	++	+	+	+	++	++	+	++	+++

Findings were scored semiquantitatively (-, not observed; +, low amounts; ++, moderate amounts; +++, high amounts). Infection groups: TH, high-dose infection with *T. gondii*; TL, low-dose infection with *T. gondii*; EH, high-dose infection with *E. tenella;* EL, low-dose infection with *E. tenella*, CI co-infected group.

^a^Et, *E. tenella*

^b^Tg, *T. gondii*

^c^Not clearly observable due to the numerous replication of *T. gondii* tachyzoites presented in cell culture by light microscopy

**Fig. 2:**
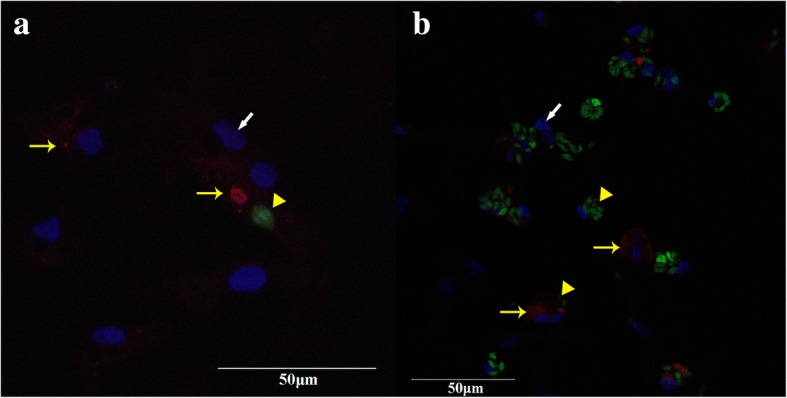
Morphological visualization during co-infection of primary macrophage cell cultures by confocal laser scanning microscopy (CLSM). In co-infected macrophages, populations of *T. gondii* partially replicated in host cells which also stained positive for *E. tenella*. Nuclei of macrophages are stained blue (DAPI), *T. gondii* RH-GFP appear green and *E. tenella* Houghton-YFP red. **a** Cell culture co-infected with *T. gondii* and *E. tenella* at 24 hpi. **b** Cell culture co-infected with *T. gondii* and *E. tenella* at 48 hpi. White arrow: macrophage nuclei; yellow arrowhead: *T. gondii* tachyzoites; yellow arrow: *E. tenella* sporozoites or meronts

In CLSM experiments using fluorescing transgenic parasites, it was observed that in co-infected macrophage cultures *T. gondii* partially replicated within host cells that also harbored *E. tenella* (Fig. 2a) from 24 hpi onwards. At 48 hpi, *T. gondii* meront numbers dominated over *E. tenella* meront numbers in the co-infected cell cultures of group CI (Fig. 2b). Host cell aggregation was seen in parallel with replication of *T. gondii* from 24 hpi.

### Assessment of parasite replication by qPCR

**Fig. 3:**
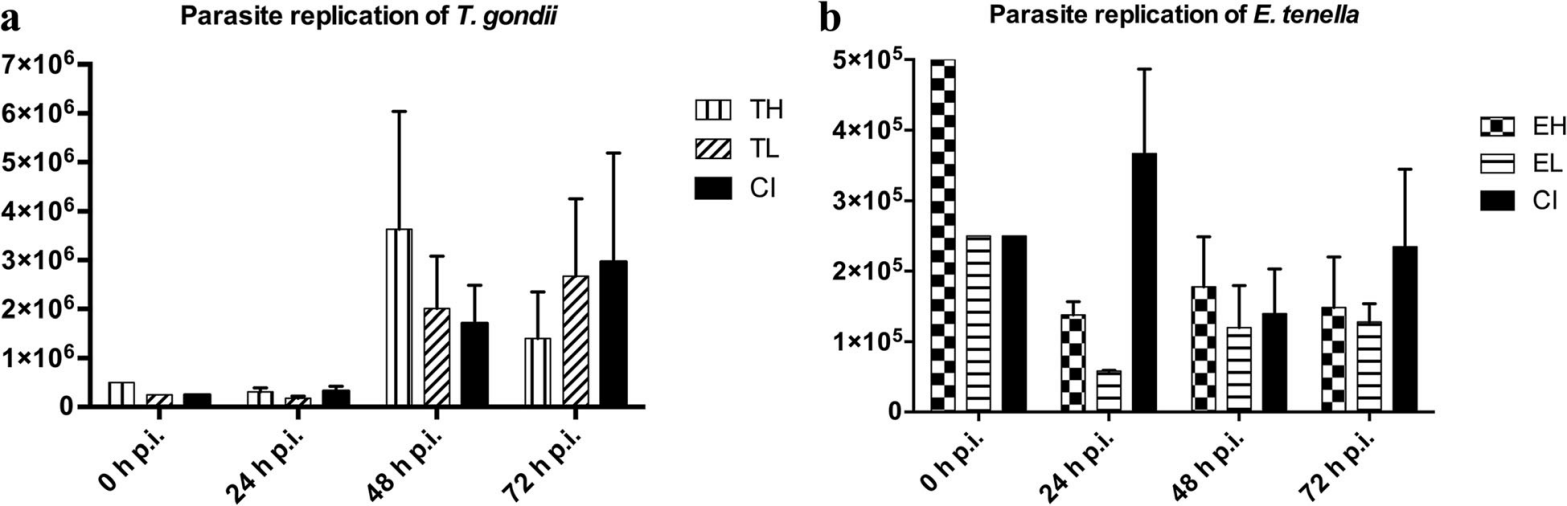
Parasite replication during infection of primary macrophage cell cultures by qPCR. Co-infection (CI) showed highest replication 72 hpi. Parasite replication is shown as mean value with standard deviation (*n* = 3). **a** *T. gondii* replication in absence (TH, TL) or presence (CI) of *E. tenella***.** *Abbreviations*: TL: low-dose *T. gondii* infection; TH high-dose *T. gondii* infection. **b** *E. tenella* replication in absence (EH, EL) or presence (CI) of *T. gondii*. *Abbreviations*: EH, high-dose *E. tenella* infection; EL: low-dose *E. tenella* infection; CI: co-infection

DNA copy quantities representing *T. gondii* tachyzoites and *E. tenella* (all stages) in all samples were measured by qPCR. The average parasite replication dynamics were different in mono-infected groups compared to the CI group for both parasites (Fig. 3). In mono-infected groups, qPCR results indicated that *T. gondii* was able to replicate considerably in infected chicken macrophages of groups TH and TL over the study period. In the TH group, a final decrease in *T. gondii* stage numbers was seen 72 h.

### Positive control MDBK cultures

Both parasites could be demonstrated in infected MDBK monolayers. Generally, in co-infected MDBK cell populations, *T. gondii* parasite numbers were slightly higher in comparison to mono-infected cultures as assessed by qPCR. However, no significant difference between mono- and co-infection was observed for the replication of both parasites (data not shown).

**Fig. 4:**
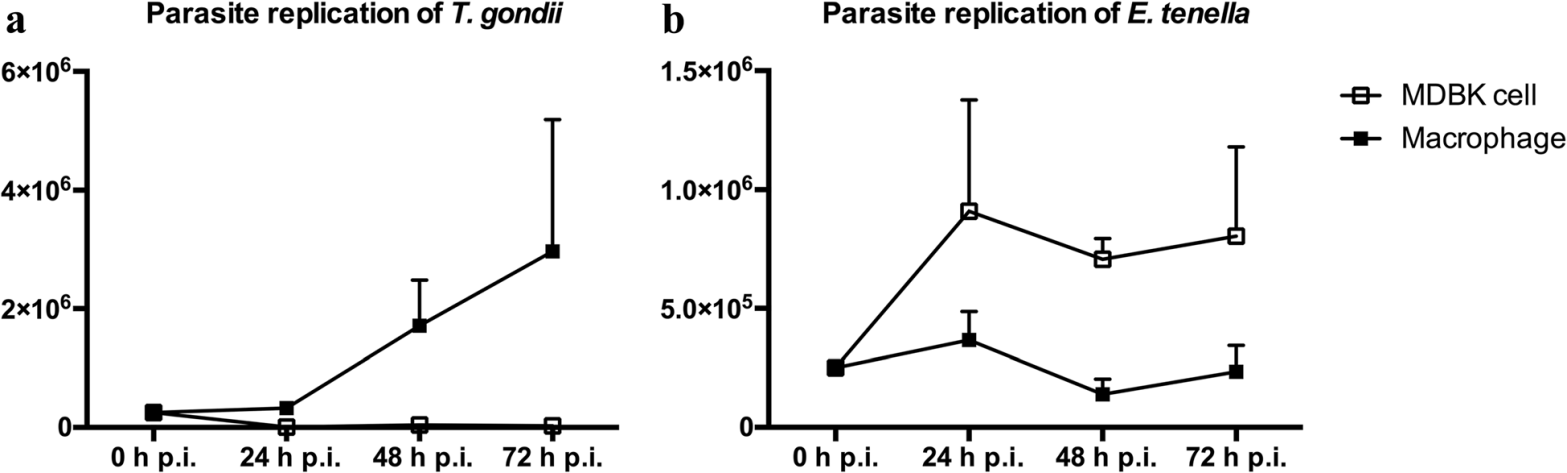
Comparison of parasite replication during co-infection (CI group) of primary macrophages cell cultures and MDBK cells. Parasite replication was significantly different for both *T. gondii* and *E. tenella* in chicken macrophages compared to MDBK. **a** Parasite replication of *T. gondii*. **b** Parasite replication of *E. tenella* (mean and standard deviation, *n* = 3)

Compared to macrophages, the stage numbers of both parasites at 72 hpi were significantly different in MDBK cells (Fig. 4). *Toxoplasma gondii* tachyzoites were not able to replicate sustainably in the MDBK co-infected culture and were not demonstrable after 24 hpi. The number of *T. gondii* tachyzoites was significantly lower in all mono- and co-infected MDBK cultures compared to the population in similarly infected macrophage cell cultures throughout the infection period. Conversely, replication of *E. tenella* was approximately quadrupled in relation to the infection dose at 24 hpi in co-infected MDBK cultures. By light microscopic observation, infected MDBK host cells showed less morphological alterations than macrophages. *Eimeria tenella* merozoites were clearly observed at 72 hpi.

### Cytokine analysis

**Fig 5:**
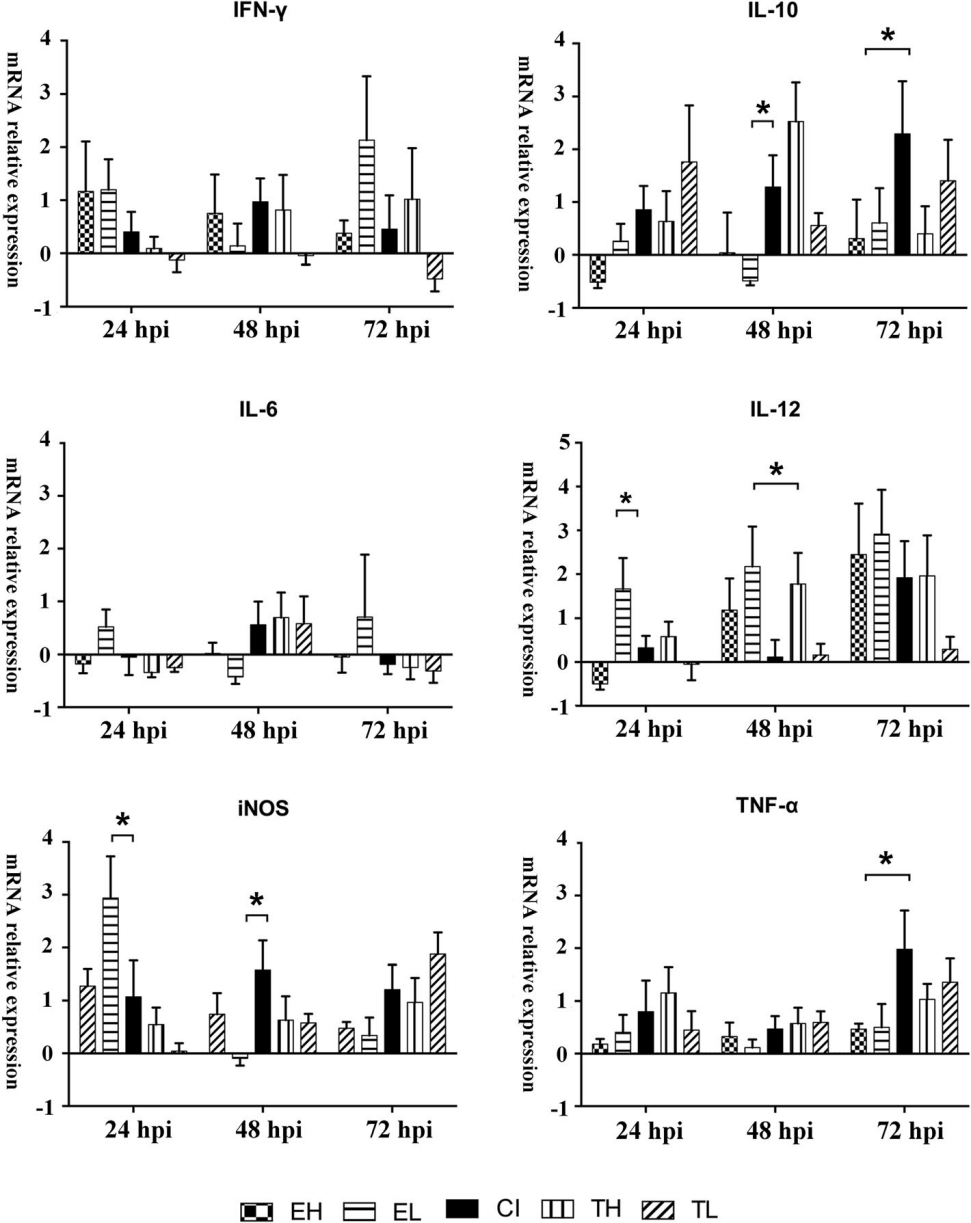
Macrophage mRNA relative cytokine expression by infection group. Normalization of data was performed on housekeeping genes GAPDH and G6PDH. The mRNA transcriptional levels of TNF-α, IL-6, IL-10, IL-12, IFN-γ, iNOS were determined by RT-qPCR. Displayed x-fold change values were calculated based on negative control (NC) group replicates (*n* = 6). Statistical comparison was performed between the CI group and other groups (*P < 0.05). *Abbreviations*: TH, high-dose infection with *T. gondii*; TL, low-dose infection with *T. gondii*; EH, high-dose infection with *E. tenella*; EL, low-dose infection with *E. tenella*; CI, co-infected group

The relative mRNA expression of six cytokines was measured until 72 hpi by qPCR (Fig. 5) and compared with the uninfected group NC as X-fold changes. From the investigated cytokine panel, only IL-6 did not show relevant alterations in any group at any time point.

The measurement at 24 hpi showed a distinct elevation for iNOS and IFN-γ mRNA expression in EH and EL as well as CI groups (*F*_(5, 31)_ = 4.81, *P* < 0.05 compared to NC group). In contrast, TNF-α was only slightly increased. For groups TH and TL, iNOS and IFN-γ were not significantly altered though TNF-α and IL-10 were slightly increased. Only in group EL, an observable increase in IL-12 expression was induced (*F*_(4, 26)_ = 3.76, *P* < 0.05 compared to all other groups). No statistically significant differences were revealed between mono-infected groups and the CI group for IFN-γ, IL-10, IL-12 or TNF-α, respectively.

At 48 hpi, IFN-γ mRNA expression was highest in group CI though group differences were not statistically significant. Significantly increased levels of IL-10 were observed in groups TH (*F*_(4, 26)_ = 4.20, *P* = 0.020 compared to TL) and CI (*F*_(4, 26)_ = 4.20, *P* = 0.025 compared to EL; *F*_(4, 26)_ = 4.20, *P* = 0.105 compared to TH). IL-12 was highly expressed in groups EH, EL, and TH. In contrast, IL-12 mRNA expression was not elevated in group CI (*F*_(4, 23)_ = 2.31, *P* = 0.026; *F*_(4, 23)_ = 2.31, *P* = 0.029 compared to EL and TH). Expression of iNOS mRNA peaked in group CI at 48 hpi (*F*_(4, 26)_ = 2.32, *P* = 0.006 compared to EL). TNF-α was not altered significantly by any infection mode until 48 hpi (*F*_(4, 24)_ = 0.656, *P* > 0.05 for all group comparisons).

At 72 hpi, IFN-γ mRNA expression levels were significantly increased for group EL (*F*_(4, 24)_ = 1.64, *P* = 0.021 compared to EH). IL-10 expression was higher in groups TH and CI (*F*_(4, 24)_ = 1.20, *P* < 0.05 compared to EH and EL). IL-12 expression was upregulated in all infected groups except for TL to a 2-fold to 3-fold extent. A moderate upregulation of iNOS as well as TNF-α expression were seen in all *T. gondii*-infected groups TH, TL and CI.

## Discussion

Investigations into physiological alterations associated with experimental infections with mixed *Eimeria* spp. in chickens were previously published [39, 40]. However, to the best of our knowledge, there is only one published study of *Eimeria* spp. co-infection with *T. gondii* [19]*,* probably because of the widely assumed sub-clinical character of toxoplasmosis in chickens [4]. The lack in primary clinical presentation does not necessarily imply that *T. gondii* infections may not influence the course of other infectious diseases in chickens. Since both pathogens, *Eimeria* spp. and *T. gondii*, are widely distributed in chicken flocks [4, 6, 41], this study focused on their potential interactions on the avian innate immune cells using primary macrophages as a model. Although the single published *in vivo* co-infection study of *T. gondii* and *E. tenella* did not demonstrate major mutual interaction in terms of inflammation and pathological findings, a significantly lower *E. tenella* oocyst excretion was observed in the co-infection group compared to *Eimeria* mono-infection group [19]. In the present *in vitro T. gondii* and *E. tenella* co-infection study, we found that there were various mutual effects between those parasites in chicken primary macrophages.

It was previously shown that chicken macrophages isolated from peritoneal exudates are capable of *E. tenella* sporozoite phagocytosis at 2–3 hpi [42]. There are convincing evidences that the clinical course and lethality of *E. tenella* infection were reduced by activated macrophages [43, 44]. On the other hand, sporozoites of *Eimeria* utilize chicken macrophages as transport cells [18], and tachyzoites of *T. gondii* can multiply in chicken primary macrophages [33, 45]. In the present study, we established a PBMC-derived macrophage culture for successful replication of both *E. tenella* and *T. gondii*. In general, *T. gondii* seemed to be better adapted to replication within chicken macrophages; however, *E. tenella* stages multiplied as well. As expected, both low-dose mono-infected groups EL and TL showed a slower and more sustainable increase in parasite numbers over time than the high-dose infected groups EH and TH. In a primary cell culture with a limited availability of host cells, it seems plausible that a high infection dose destroys host cells faster; thus rapidly hampering parasites replication than a low initial number of parasites. Although *E. tenella* showed an initial decrease in total gene copy numbers, this was followed by a distinct increase in co-infected cultures only. This may reflect that *T. gondii* supports *E. tenella* replication in macrophages; however, this hypothesis needs further investigation.

Mixed *Eimeria* spp. infections *in vivo* did not exert mutual effects on replication of three poultry *Eimeria* species [46]. In contrast, in our *in vitro* model we found that *E. tenella* and *T. gondii* displayed interactions that were most pronounced towards the end of the study (72 hpi). At this time, both *T. gondii* and *E. tenella* were replicating more strongly in co-infected cultures than in the mono-infected controls. This effect was particularly pronounced for *E. tenella*. However, the capability of *E. tenella* to invade macrophages was not influenced by co-infection with *T. gondii*. Light microscopic observations showed that first generation meronts were the dominating stages of *E. tenella* in all mono- and co-infected cultures. Unfortunately, the chosen co-infection model is not able to investigate a potential influence of *T. gondii* on sexual *E. tenella* development. It can be speculated that the increase in asexual *E. tenella* seen during co-infection might only be temporary and stage-related since another recent *in vivo* investigation in our laboratory showed that a significantly low number of *E. tenella* oocysts were excreted by chicken co-infected with *T. gondii* than following *Eimeria* mono-infections [19]. In accord with our current findings, Hiob et al. [19] stated that the number of meronts in the intestine was not significantly altered by co-infection so the inhibiting effect of *T. gondii* on chicken *Eimeria* might be rather linked to the sexual development, which is not described to take place in macrophages.

The MDBK cell line was used to investigate growth and functions of *T. gondii* and *E. tenella in vitro* in various ways [47, 48]. Regardless of mono- or co-infection, we found that reproduction of *T. gondii* tachyzoites was broadly lower in MDBK cells than in chicken primary macrophages. In contrast to *E. tenella*, *T. gondii* replicated much more strongly in the macrophage culture. It appears possible that the cell metabolism of MDBK cells is altered at 41 °C, which is distinctly above the physiological bovine body temperature. However, no significant effect on parasite multiplication was found in co-infected MDBK cell cultures compared to primary chicken macrophages. This indicates an important role of the host cell type in induction or modulation of pathogen-pathogen interactions whereas the incubation temperature of 41 °C does not appear to play a major role in our model.

Chicken macrophages serve as phagocytes and regulatory immune cells. They produce cytokines, induce cytokine production in other immune cells, and destroy protozoans directly as part of the innate immune response [49, 50]. Although chickens are considered to be important natural hosts for *T. gondii* [4] and the only host species for *E. tenella* [51], immunoregulatory mechanisms by avian primary macrophages during simultaneous *T. gondii* and *E. tenella* infections are not sufficiently elucidated. We could demonstrate that, besides affecting the parasite replication potential, primary macrophage cell cultures reacted in different ways to both parasites during mono- and co-infections.

Macrophages are not the main source of IFN-γ but they are capable of IFN-γ expression [52]. IFN-γ plays an important role in the replication inhibition of the two investigated parasites [53, 54]. Five other cytokines produced by macrophages were included in our investigation: Th2-supporting IL-6, Th1-supporting IL-12, IFN-γ-inhibiting IL-10, and innate immune response-related iNOS and TNF-α. This panel was chosen because earlier studies clearly indicated the importance of those cytokines in coccidial infections [31, 46]. Single infections with *E. tenella* evoked host immune response which led to significant expression of cytokines such as IL-10 and IFN-γ in the ceca [46]. *In vivo*, it was demonstrated that mRNA expression of IFN-γ, IL-12 and IL-10 was distinctly upregulated in the ceca of chickens at the early stage of co-infection with *T. gondii* and *Eimeria* spp. [19]. In addition, high expression of those cytokines was observed in chickens co-infected with *E. tenella* and *Clostridium perfringens* [55]. Based on our observations in the co-infection model, using 12 hour intervals for cytokine expression measurements might be useful in the future to more comprehensively judge parasite-host cell interactions. However, we could observe several host cell reactions in this co-infected model.

In the present study, IFN-γ mRNA levels were upregulated mainly due to *E. tenella* infection, whereas co-infection seemed to suppress this Th1-related cytokine. Similar findings were recorded for IL-12 mRNA expression. Thus, it can be assumed that the adaptive Th1 response is rather suppressed than triggered in co-infected macrophages compared to mono-infections. In a recent study [56], a significant upregulation at 2 hpi and slight downregulation of IL-12 at 24 hpi were observed in a chicken macrophage cell line infected with *E. tenella* merozoites, which was diminishing over time. In contrast, IL-12 was upregulated by the end of the observation period in group CI, which might be related to the development of *E. tenella* meronts. Interestingly, in contrast to the cited *in vivo* studies [19, 55], we did not observe a distinct increase in IL-12 mRNA expression in co-infected macrophages. Therefore, we assume that not macrophages but other immune cell populations are responsible for the previously observed *in vivo* increase of IL-12 expression. In chickens, IL-6 mRNA expression was not significantly affected. This indicates that Th2 stimulation by macrophage-derived IL-6 does not play a major role in this infection mode.

IL-10 mRNA upregulation was observed mainly in T. *gondii* infection with a delayed peak in group CI that coincides with increased *E. tenella* replication. Since one of the multiple functions of IL-10 is the downregulation of IFN-γ expression, this corresponds clearly with the reduced transcription seen in CI. However, high expression of IL-10 in infected chickens is not associated to parasite multiplication or expression of cytokines like IFN-γ and IL-12 during *T. gondii* RH-infection [57]. Thus, it seems as if *in vivo* multiple immune cell populations influence the Th1 response to apicomplexan parasites, with macrophages being an important part of the complex innate immune response.

In spite of the relatively low IFN-γ levels in co-infected macrophages, reactive oxygen and nitrogen intermediates such as iNOS were produced in excess in these cells. This is interesting because IFN-γ is generally assumed to trigger the macrophages to produce these metabolites [31, 58]. It was previously [59, 60] demonstrated that *T. gondii* is altering NO production inhibition in chicken monocyte-derived macrophages and macrophage cell lines. In the present study, iNOS seemed to play a vital role in macrophage response during co-infection (Fig. 5). Conversely, it was reported that iNOS was distinctly expressed during *E. tenella* infection *in vivo* or *ex vivo* [29]. This is in line with the general but moderate upregulation of iNOS mRNA expression in all infected groups seen in the present study.

Furthermore, we observed increased TNF-α expression in all infected groups, and this was particularly distinct in *T. gondii*-infected groups reaching a maximum at 72 hpi in the co-infected group. TNF-α increase was described before in *E. tenella*-infected chicken macrophages [32, 61]. Other authors [62, 63] stated a synergistic anti-*T. gondii* effect of TNF-α and IFN-γ. Our study demonstrated that co-infection significantly upregulated TNF-α while parasite replication increased more than in mono-infected cultures. We conclude that TNF-α production may either not be sufficient to counteract parasite replication in our model, especially in the presence of low IFN-γ levels, as indicated by Chang et al. [63], or that TNF-α mRNA expression does not exactly reflect actual TNF-α levels.

## Conclusions

We demonstrated *in vitro* interactions between *T. gondii* and *E. tenella* in macrophages and MDBK cells. The findings of this study revealed that over a study period of 72 hpi, the replication of both parasites increased during co-infections compared to mono-infected cultures. Increased expression of IL-10 and TNF-ɑ in co-infected cells demonstrates that interaction of both parasites is tightly linked to the host cell types and their various responses to infection. However, the present study leads to further questions. Additional experiments are needed to fully clarify the signaling pathways that are, e.g. leading to replication differences between mono- and co-infected cells. The presented findings are currently based solely on *in vitro* experiments that were chosen because of the defined conditions that allow for a more precise initial data interpretation. Future *in vivo* studies taking into account the natural interactions between different immune cell populations are needed to confirm our findings and their biological relevance, as well as to enable a betterunderstanding of the mechanisms of host-parasite and parasite-parasite interactions during co-infections. Additionally, investigations into subsequent infections with both pathogens will be helpful to estimate the relevance of non-simultaneous infections with both parasites that might be most relevant in the field.

## Abbreviations

iNOS: inducible nitric oxide synthase
IL: Interleukin
IFN-γ: Interferon gamma
TNF-α: Tumor necrosis factor-α
TH: High-dose infection with *T. gondii*
TL: Low-dose infection with *T. gondii*
EH: High-dose infection with *E. tenella*
EL: Low-dose infection with *E. tenella*
CI: Co-infected group
hpi: hours post-infection
NC: Negative control group
MDBK: Madin-Darby Bovine Kidney
PBMC: Peripheral blood mononuclear cells
PBS: Phosphate-buffered saline
IFA: Immune fluorescence assay
CLSM: Confocal laser scanning microscopy
